# Readiness of nurses when faced with a patient’s death

**DOI:** 10.3389/fpubh.2024.1399025

**Published:** 2024-08-02

**Authors:** Marta Kowalenko, Elżbieta Krajewska-Kułak, Beata Kowalewska, Agnieszka Kułak-Bejda, Teresa Kulik, Aleksandra Gaworska-Krzemińska, Katarzyna Van Damme-Ostapowicz

**Affiliations:** ^1^Universitas Cardinalis Stephani Wyszyński Varsoviae, Cardinal Stefan Wyszyński University in Warsaw, Warsaw, Poland; ^2^Department of Integrated Medical Care, Medical University of Białystok, Białystok, Poland; ^3^Department of Psychiatry, Medical University of Bialystok, Białystok, Poland; ^4^State University of Applied Sciences in Krosno, Krosno, Poland; ^5^Institute of Nursing and Midwifery, Medical University of Gdansk, Gdansk, Poland; ^6^Western Norway University of Applied Sciences, Faculty of Health and Social Sciences, Førde, Norway

**Keywords:** nursing, death, patients, SWLS, CISS, GSES, CECS

## Abstract

**Introduction:**

The death of a patient negatively affects the professional dimension of nurses’ functioning and also their private lives, where professional experiences and emotions are often transmitted.

**Aim of the study:**

The main aim of the study was to discover how the nursing staff assessed their self-efficacy in dealing with the death of a patient.

**Materials and methods:**

The researchers used a diagnostic survey method and a self-authored survey, Life Satisfaction Scale (SWLS), Coping Inventory for Stressful Situations (CISS), Generalized Self-Efficacy Scale (GSES), Courtauld Emotional Control Scale (CECS), The Death Anxiety and Fascination Scale with the Death Anxiety Subscale. The study group consisted of 287 individuals.

**Results:**

Working in a hospital is stressful in the opinion of 79.44% of the respondents. 39.37% of the respondents feared death. In the course of work, the death of a patient in the department was sometimes experienced by 34.84% of the respondents, always experienced by 29.97%. The respondents usually did not make their compassion for a dying patient based on the patient’s position in society (57.84%). In the case of the majority (84.67%) of the respondents, the employer did not provide mental support for the staff in difficult situations. The majority of the respondents did not feel the need to broaden their knowledge of death and dying (64.11%).

**Conclusion:**

The surveyed nurses most often presented low or average life satisfaction, high self-efficacy, average levels of stress and coping in all three styles (with a preference for avoidance-oriented), and a high level of death fascination and an average level of death anxiety. The majority of the nurses in difficult situations and when a stressful situation occurred did not receive support from their employer or from physicians, but could count on the assistance of fellow nurses and a divisional nurse. The majority of the respondents felt that psychological support in their work was important and would gladly benefit from it. Although most nurses did not feel the need to broaden their knowledge of death/dying, they would take part in such a course if it were possible.

## Introduction

In nursing, the death of a patient is considered a common professional situation, which provokes not only desirable emotions, such as composure and calmness but also grief and hopelessness, which is why nurses may be more likely to experience anxiety and destructive effects of negative feelings. The death of a patient negatively affects the professional dimension of nurses’ functioning and also their private lives, where professional experiences and emotions are often transmitted (1).

Many researchers have tried to systematize human attitudes when dealing with a dying person. There are several patterns of behavior. The first one is a negative pattern—objectification of a dying person and treating them like a thing (2–5). Another pattern is expressed through an individualistic approach (the dying process is a private affair of an individual, who should not burden their community with difficult experiences and their emotions or expect care in the last moments before death) (2–4). The pattern opposite to the individualism pattern is personalism (4). A completely different, philosophical and heterocentric attitude is often presented by religious people. From this perspective, the main task of a nurse while caring for a patient is to make them feel needed (6). Szaniawski (7) distinguishes five key attitudes toward death, depending on personality: ambivalent, calm, religious, evasive, and terrified. There are also three factors that determine the attitudes of nurses and midwives toward a dying patient, namely, emotions, behaviors, and experiences, which may have a negative impact on caring for patients. They may be manifested through treating a patient instrumentally, ignoring their needs, but also positively, by responding to a patient’s needs with respect for all their rights (8–10). Due to the job specifics and working conditions, imposing a heavy psychological burden resulting from frequent contact with dying patients, the nursing staff are particularly vulnerable to chronic fatigue. Close encounters with dying patients widely broadens nurses’ experience but, at the same time, requires them to be well-prepared and have the right attitude. Unlike chronic physical tiredness, the latter is subjective in nature, which, according to John Swaller’s concept, means that a person experiences it under cognitive load. It may be manifested through such symptoms as nervous excitability, reduced work effectiveness, thought and/or concentration disturbances, insomnia, and even decreased libido (11).

Self-efficacy refers to an individual’s perception of their ability to perform specific behaviors (12). Albert Bandura’s self-efficacy theory was intended to unify successful coping and goal achievement and focused on outcome and efficacy expectations as the key ingredients. In the nursing context, self-efficacy plays a crucial role in promoting emotional and behavioral changes related to health problems. It encompasses cognitive, motivational, affective, and selection processes (13). Self-efficacy influences how nursing staff cope with challenging situations, including patient deaths. When assessing self-efficacy in nursing staff dealing with patient death, researchers may encounter gaps related to specific contexts, measurement tools, or interventions.

Death-coping self-efficacy (DCS) measures a nurse’s confidence in providing palliative care to dying patients and their families (14). It involves being confident in assessing needs, solving care issues, managing symptoms and providing information, handling the grief of losing someone, and arranging for burial.

Few studies have been conducted on self-efficacy in nurses in the context of death.

A Taiwanese study by Hsu and Tsau (14) of 556 university nursing students showed that high levels of openness, agreeableness, and conscientiousness, as well as low neuroticism, were associated with the ability to talk about death, the ability to process loss, the intention to increase meaning in life, and the ability to process funeral practices. The authors suggested that high levels of neuroticism and task-oriented coping strategies are less effective in dealing with significant loss or the death of a significant other.

Lin et al. (15) conducted a study with 572 Chinese nurses to examine how different personality profiles relate to clinical nurses’ ability to cope with death in general wards and ICU. Among the Big Five Personality Traits, in nurses the score was highest for conscientiousness and lowest for neuroticism. Openness, agreeableness, and conscientiousness were significantly associated with DCS in nurses.

A Polish study from 2021 (16) analyzed the feelings and emotions which accompany nurses during their work when they face the death of patients in a surgery unit, an internal medicine unit, a hospital emergency department, and an intensive care unit. The authors used Mini-COPE and PSS-10 questionnaires to assess the level of anxiety and the ways of coping with the stress of nurses related to contact with dying patients. Compassion, sadness, and helplessness were the most common types of nurses’ emotions caused by the death of patients, regardless of the nurses’ length of service and the place of work. The way of coping with stress is related to the period of service and the workplace of nurses.

## Aim of the study

The main aim of the study was to evaluate how the nursing staff assessed their self-efficacy in dealing with the death of a patient, to learn about their level of preparedness in this regard, and to assess the emotional control and coping skills presented in a difficult situation. The following were considered as specific goals: to learn about the attitudes of the nursing staff toward dying patients, to reveal the emotions and experiences accompanying the nursing staff in case of a patient’s death, to assess the impact of the death of patients on the personal lives of the respondents, a degree of coping in crisis situations, the nursing staff’s knowledge of stress-relieving methods, and to assess whether there was a need for workshops with psychologists, psychiatrists on coping with the deaths of patients and how to talk abou them with the family.

The following research hypotheses were set: 1. The nursing staff are rather dissatisfied with their lives, whereas their life satisfaction depends on their age, length of service and place of practice. 2. The nursing staff have a high stress level, depending on the age of the respondents, length of service and place of practice, and prefer avoidance-oriented coping. 3. When a difficult/stressful situation arises, the nursing staff can count on the assistance of the employer and other members of the therapeutic team. 4. The nursing staff assess their self-efficacy as high. 5. The nursing staff have a high level of death fascination and anxiety. 6. The nursing staff does not make showing a dying patient compassion dependent on the patient’s position in society, nor is it influenced by the study subjects’ age, job specifics, length of service, life satisfaction, stress coping style, and level of emotional control. 7. Perceiving the death of patients from different age groups as harrowing is not affected by the age of the respondents, their length of service, place of practice, life satisfaction, stress coping style, or level of emotional control. 8. The nursing staff show interest in broadening their knowledge of how to deal with the death of patients.

## Materials and methods

There are 239,257 nurses employed in Poland (data as of 31 December 2023). The sample size was calculated from the group of employed nurses with a confidence level of 95% and a maximum error of 8%, and it was set at a minimum of 150 people. The study group consists of 287 nurses, so the criteria were met.

The study was conducted after obtaining consent from the Bioethics Committee R-I-002/631/2019, the Medical University of Bialystok, Provincial Specialist Hospital in Biała Podlaska, Children’s Memorial Health Institute in Warsaw, Independent Public Hospital No. 4 in Lublin, and Ludwig Zamenhoff University Children Clinical Hospital in Białystok.

Participation was voluntary and anonymous. Participants could withdraw from or leave the study at any point without feeling obligated to continue. Personally identifiable data was not collected. Using a convenient sampling method, envelopes containing a questionnaire and a declaration of written consent were provided to each participant. The aim of the study was explained and the voluntary and confidential nature of this research was emphasized.

The researchers used a diagnostic survey method, and the main study was preceded by a pilot study involving a group of 50 individuals to check if the questions contained in the survey were clear. The main study, lasting from 12/09/2019 to 04/02/2020, involved 287 subjects working in the nursing profession. Sample selection was purposive. 300 questionnaires were distributed, of which 287 were returned. The response rate was 95.6%/ After the respondents completed the questionnaires, the authors of the study transferred the questionnaires to the principal investigator completed questionnaires.

The researchers used a set of questionnaires:

- a self-authored survey: 49 questions
- FAS (Family Affluence Scale) consisting of four questions: Does your family own a car, van or truck? Do you have your own bedroom for yourself? During the past 12 months, how many times did you travel away on holiday with your family? How many computers does your family own? “No” is scored 0 points, “yes” is scored 1 point, “two and more” is scored 2 points. FAS is considered: very low when a score is 0–1 points; low is 2–3 points; average is 4–5 points; and high is above 6 points (17).
- SWLS – Life Satisfaction Scale, *by Diener, Emmons, Larsen, Griffin, a Polish version by Juczyński*, for other professionals who are not psychologists. It consists of five statements. The respondent rated how each statement applied to their life to date, where 1 meant “definitely disagree,” and 7 “definitely agree.” The subject’s scores were summed up, and the overall result described the level of life satisfaction. The scores could range from 5 to 35 points, with higher values corresponding to higher life satisfaction. In interpreting the score, a sten scale was also used; the score of 1–4 sten was regarded as low, 7–10 sten as high, and 5 and 6 sten as average. The reliability index (*Cronbach’s* alpha) of *SWLS,* determined in a study involving 371 adults, is 0.81. The scale constancy index, determined in a double study involving a group of 30 individuals 6 weeks apart, was 0.86 (18).
- CISS *(Coping Inventory for Stressful Situations)—* (18) Polish version (19): The questionnaire is used to study stress coping styles: task-oriented coping, emotion-oriented coping, and avoidance-oriented coping. Each aspect of a stress coping style consists of 16 items; the subjects can obtain from 16 to 80 points in each of them. The results obtained were also presented on a sten scale, which consists of 10 units (sten scores). One unit equals 0.5 of standard deviation. Each scale unit corresponds to a certain percentage of the area under the normal curve of the score distribution. Scores within the range of 5–6 sten are regarded as average, within the range of 7–10 sten as high, and within the range of 1–4 sten as low. The reliability of the Polish version of the *CISS* questionnaire, as measured by *Cronbach’s alpha* coefficient, ranges from 0.72 to 0.92 (14).
- GSES (*Generalized Self-Efficacy Scale*) (20) is consisting of 10 statements. It measures the respondents’ general belief in their ability to respond to difficult situations and obstacles. The respondent selects their answers by circling the appropriate number. There are four answers to choose from for each question: not at all true – 1, hardly true – 2, moderately true – 3, and exactly true −4. The total score reflects an overall self-efficacy index, which can range from 10 to 40 points. High scores represent high self-efficacy. After transformation into standardized units, a general indicator was interpreted according to the characteristics of the sten scale. Results within: A sten score of 1–4 was regarded as low, a sten score of 7–1 0 as high, and a sten score of 5–6 as average. The Cronbach’s alpha coefficient was 0.85. The reliability of the scale assessed by test–retest (after 5 weeks) was 0.78.
- CECS *(Courtauld Emotional Control Scale)* (20). It is used to measure subjective control of anger, anxiety, and depression in difficult situations. It consists of three subscales, each including seven statements about the expression of anger, depression, and anxiety. The respondent determined the frequency of a particular method of expressing their emotions on a four-point scale, ranging from “almost never” – 1 point to “almost always” – 4 points. The scores for each of the three subscales range from 7 to 28 points. The overall emotional control index ranges from 21 to 84 points. The higher the score, the greater the suppression of negative emotions. The reliability of the scale was assessed by estimating its internal consistency and absolute stability—Cronbach’s alpha coefficients: for anger control: 0.80; depression: 0.77; anxiety: 0.78; and for the total emotional control index: 0.87.
- The Death Anxiety and Fascination Scale with the Death Anxiety Subscale (21), The test consists of 23 items and two scales; the responses range between 1 and 4 points. The Death Anxiety Scale, consisting of nine questions, is used to measure a conscious level of death anxiety, especially in relation to one’s own death: the higher the death anxiety, the more a cruel, dark, and dirty death appears; Cronbach’s α = 0.80. It was assumed that ≤17 points means a low level of anxiety, 18 points an average level of anxiety, and 19–36 points a high level of anxiety. The Death Fascination Scale: consists of 14 items and measures death fascination as a cognitive interest in the topic of death and dying. The score of this scale was not considered in this study. The higher the fascination, the more pure and merciful death seems; Cronbach’s α = 0.90. A score of ≤27 points was regarded as a low level of fascination, 28 points as an average level of fascination, and 29–56 points as a high level of fascination.

The study’s inclusion criteria were active nursing practice, at least 1 year of service, and consent to participate. The exclusion criteria were failure to meet the above criteria.

### Statistical analysis

Before analyzing the data, the Shapiro–Wilk test was used to test normality. Statistical analysis was performed using R studio software and the R programming language. In the comparisons, analysis of variance (ANOVA) with Bonferroni Holm’s *post-hoc* test was used for quantitative variables with a distribution close to normal, and Kruskal-Wallis tests with a *post-hoc* test of multiple comparisons for quantitative variables with a distribution different from normal. Correlation assessment was performed using Kendall’s Tau correlation coefficient due to the ordinal nature of the scales. The relationship between the questionnaire results and the selected questions was assessed with a general linear model, taking into account the influence of age, length of service, and place of work as possible confounding variables.

The study group comprised 287 individuals, including 91.64% women and 8.36% men. Most respondents were aged between 41 and 60 (67.6%). More than a third (37.98%) lived in cities with a population of more than 50,000. The remaining respondents lived in smaller towns (43.91%) or rural areas (18.12%). More than half of the respondents were married (65.16%) and had a university degree (69.69%). Other results are shown in Table 1.

**Table 1:** Characteristics of the study group.

	*n*	%
Sex		
Female	263	91.64%
Male	24	8.36%
Age		
20–30 years old	47	16.38%
31–40 years old	36	12.54%
41–50 years old	98	34.15%
51–60 years old	96	33.45%
>60 years old	10	3.48%
Place of residence		
Rural area	52	18.12%
Town with a population below 5,000	34	11.85%
Town with a population of 5,000–20,000	38	13.24%
Town with a population of 21,000–50,000	54	18.82%
Town with a population of 50,000–40,000	109	37.98%
Marital status		
Single	53	18.47%
Married	187	65.16%
Divorcee	36	12.54%
Widow/widower	6	2.09%
Cohabitating	5	1.74%
Education		
Secondary	41	14.29%
Post-secondary	46	16.03%
University degree	200	69.69%

The respondents’ specialties included: surgery (11.67%), anaesthesiology (9.67%), oncology (5.67%), gynecology (3.00%), geriatrics (1.33%), cardiology (1.00%), internal medicine and neonatology (0.33% each). A specialty in oncology with palliative care was declared by 1% of the respondents, and a specialty in extended-term care and palliative care—by 0.33% each. The remaining part of the study group had no specialty. The oncology qualification course had been completed by 1.00% of nurses, the extended-term care course by 1.67%, the course in caring for a child with cancer by 1.33%, and the specialist cancer course by 0.33%. Most respondents had more than 25 years of service (48.78%). The subsequent groups: 21–25 years (12.89%), 16–20 years (8.01%), 11–15 years (6.97%), 6–10 years (4.88%), 1–5 years (11.85%) and less than a year (6.62%). The majority of the respondents (77.00%) were employed in hospitals for adult patients and 23.00% in pediatric hospitals. Over half (56.79%) of the respondents worked with adults, 14.98% with the older adult, 24.39% with children, and 3.83% with adolescents.

Based on the FAS scale, the researchers found that the majority of the respondents (81.18%) had an average affluence. The remaining part could be regarded as relatively affluent (14.29%), very affluent (2.44%) and relatively poor (2.09%). The majority of the respondents were Catholic (91.99%), while the remaining part included Orthodox (3.83%), Lutheran (1.39%), or Jehovah’s Witnesses (0.70%). Followers of other religions accounted for 0.35%, and non-believers for 1.74%. However, only 72.47% of the respondents considered themselves believers and practitioners.

## Results

Working in a hospital is stressful in the opinion of 79.44% of the respondents. 2.09% of the respondents had a different opinion, and 18.47% said it varied. When the death of a patient happened, the respondents could not count on the assistance of physicians (56.45%), but received support from fellow nurses (90.24%) and a divisional nurse (58.89%). When coping with the difficulties of experiencing a patient’s death, the respondents got help from their family (44.25%), prayer (33.80%), or a form of entertainment (18.47%). 3.48% of the individuals (8.3%) had difficulty answering the question. The most common methods that the respondents used to cope with stress were talking and meeting with friends (40.42%), shopping (22.65%), physical activity (15.33%), eating sweets (9.76%), taking medication (7.67%), and using stimulants (3.83%). Hobbies outside of work were pursued by 60.98% of the individuals. In their free time, the study subjects tended to rest actively (32.75%), sleep (22.65%), or read (16.72%). However, there were also those who managed their household and took care of children (13.94%) or worked a second job (8.71%). 57.84% of the respondents devoted 1 or 2 h to rest, 20.56% rested for less than an hour, 0.41% for 3 to 5 h, and 2.44% for more than 5 h. As many as 9.76% of the respondents said they had no free time.

39.37% of the respondents feared death, 22.30% did not fear it, and 38.33% of the respondents had difficulty answering the question. The respondents tended to think about their own death (67.25%) and the death of their loved ones (78.40%), while they tended not to think about the death of their friends (50.52%) or strangers (56.79%). In the opinion of 33.10% of the respondents, death should not always be a consequence of an illness and affect only the older adult. 28.22% of the respondents had a different opinion, while 38.68% of the respondents had a problem with giving an unequivocal answer. 54.01% of the respondents considered death as a natural process, like birth, with something unpleasant. It was considered the end of suffering and pain by 31.71% of the respondents, and a defeat in the struggle for life by 12.24%. 1.05% of the respondents had difficulty answering the question. The word “death” was usually associated by the respondents with passing away and inevitability (56.33%), with old age (42.00%), with something unpleasant (41.33%), with pain (22.67%), with illness (22.00%), with suffering (17.67%), with a transition from earthly life to the life eternal to God (10.00%), with anger or the end of problems (6.67% each), with the end of earthly life (5.33%) an oasis with peace or fear (5.00% each) and something positive (0.33%). A coffin was associated by 8.33% of the respondents with death, and by 0.33% with a cemetery. 22.33% of the respondents had difficulty answering the question. 75.61% of the respondents did not believe in life after death.

In the course of work, the death of a patient in the department was sometimes experienced by 34.84% of the respondents, always experienced by 29.97%, rarely experienced by 14.63%, and never experienced by 20.55% of the respondents. 80.14% of the respondents experienced a situation in which a patient whom they cared for died. The death of a patient particularly affected 52.96% of the respondents: in the case of 44.25%, it was the death of children; for 14.63%, the death of a young patient; for 3.14%, the death of a sick adult, and for 37.98% any kind of death. 67.60% of the respondents experienced some emotions after the death of their patient. 18.82% did not remember it, and 5.57% had no such experience. 8.01% of the nurses had problems answering the question. At the time of a patient’s death, the respondents usually felt helplessness and powerlessness (63.33%), sadness (55.00%), compassion (53.67%), depression (41.00%), grief (29.00%), emptiness (8.33%), anxiety.

(4.67%), peace (4.33%), indifference (3.67%), despair (3%), weariness (2.67%) and anger (2.00%). 0.67% of the individuals (8.3%) had difficulty answering the question.

The respondents usually did not make their compassion for a dying patient based on the patient’s position in society (57.84%); however, 27.53% admitted that they subconsciously had less compassion for certain patients, such as alcoholics or perpetrators of violence, and in 11.15% of the respondents said that “it varies.” 3.48% of the individuals (8.3%) had difficulty answering the question.

The majority (75.26%) of the respondents did not react to the death of a stranger as they would react to the death of a close person. However, 7.67% of the respondents reacted in the same way, and 17.07% had difficulty answering the question. Only 17.07% of the respondents happened to be offended by the family of a dying patient. 60.28% of the respondents had no such contact, and 22.65% did not make a clear statement on this issue.

Table 2 shows the results of the questionnaires completed by the respondents. It turned out that the respondents most often presented low life satisfaction, an average level of stress coping in all three styles (including the highest for the avoidance-oriented coping), a fairly high level of self-efficacy, a high level of fascination with death, and an average level of anxiety.

**Table 2:** Descriptive statistics of SWLS, CISS, CESS, and SLIF scores.

Variable	N	Min	Max	Median	Q1	Q3	Mean	SD
SWLS sten	287	1	9	5	3.5	6	4.53	1.7
CISS SSZ sten score	287	1	10	5	4	6	5.03	1.87
CISS SSE sten score	287	1	9	6	4	7	5.44	1.88
CISS SSU sten score	287	1	10	6	4.5	7	5.5	1.87
CISS SSU ACz sten score	287	1	10	6	5	7	5.69	1.96
CISS SSU PKT sten score	287	1	10	5	4	6	5.13	1.86
GSES sten score	287	1	10	7	6	7	6.41	1.53
CECS anger scale	287	7	28	17	14	20.5	17.3	4.49
CECS depression scale	287	7	28	18	15	20	17.5	4.01
CECS anxiety scale	287	7	28	18	16	20	18.1	3.8
SLIFS total	287	23	92	47	42	50	46.9	8.81
SLIFS death fascination subscale	287	11	44	20	18	23	20.7	4.78
SLIFS death anxiety subscale	287	9	36	18	16	20	18.4	3.75

SWLS	low	66	23	28	11	128	44.6%
	average	68	13	32	10	123	42.9%
	high	18	8	9	1	36	12.5%
CISS SSZ	low	57	15	26	9	107	37.3%
	average	66	21	33	8	128	44.6%
	high	29	8	10	5	52	18.1%
CISS SSE	low	37	14	15	9	75	26.1%
	average	70	18	40	8	136	47.4%
	high	45	12	14	5	76	26.5%
CISS SSU	low	39	15	12	6	72	25.1%
	average	67	18	38	8	131	45.6%
	high	46	11	19	8	84	29.3%
CISS SSU ACZ	low	29	12	10	7	58	20.2%
	average	65	18	31	7	121	42.2%
	high	58	14	28	8	108	37.6%
CISS SSU PKT	low	49	19	16	3	87	30.3%
	average	78	17	34	14	143	49.8%
	high	25	8	19	5	57	19.9%
GSES	low	14	9	6	0	29	10.1%
	average	47	14	23	10	94	32.8%
	high	91	21	40	12	164	57.1%

SWLS, Life Satisfaction; CESS, Emotional Control Level; CISS, Stress Coping Style; CISS SSZ, Task-oriented Coping; CISSSSE, Emotion-oriented Coping. CISS SSU, Avoidance-oriented Coping; CISSSUACZ, Avoidance-oriented Coping, Engaging in Substitute Activities; CISSSSUPKT, Avoidance-oriented Coping, Seeking Social Contacts. GSES, Self-Efficacy; SLIFS, Death Anxiety and Fascination Scale.

There were no significant relationships between the place and type of hospital where the respondents worked and their level of life satisfaction, stress coping style, self-efficacy and level of emotional control (Table 3).

**Table 3:** Comparison of questionnaire results, considering the place of work (chi-square test).

Variable		Place of residence								*p*	Cramer’s V
		Biała Podlaska		Białystok		Lublin		Warsaw			
SWLS	low	66	43.42%	23	52.27%	28	40.58%	11	50.00%	0.480	0.098
	average	68	44.74%	13	29.55%	32	46.38%	10	45.45%		
	high	18	11.84%	8	18.18%	9	13.04%	1	4.55%		
CISS SSZ	low	57	37.50%	15	34.09%	26	37.68%	9	40.91%	0.947	0.054
	average	66	43.42%	21	47.73%	33	47.83%	8	36.36%		
	high	29	19.08%	8	18.18%	10	14.49%	5	22.73%		
CISS SSE	low	37	24.34%	14	31.82%	15	21.74%	9	40.91%	0.285	0.114
	average	70	46.05%	18	40.91%	40	57.97%	8	36.36%		
	high	45	29.61%	12	27.27%	14	20.29%	5	22.73%		
CISS SSU	low	39	25.66%	15	34.09%	12	17.39%	6	27.27%	0.430	0.102
	average	67	44.08%	18	40.91%	38	55.07%	8	36.36%		
	high	46	30.26%	11	25.00%	19	27.54%	8	36.36%		
CISS SSU ACZ	low	29	19.08%	12	27.27%	10	14.49%	7	31.82%	0.537	0.094
	average	65	42.76%	18	40.91%	31	44.93%	7	31.82%		
	high	58	38.16%	14	31.82%	28	40.58%	8	36.36%		
CISS SSU PKT	low	49	32.24%	19	43.18%	16	23.19%	3	13.64%	0.092	0.138
	average	78	51.32%	17	38.64%	34	49.28%	14	63.64%		
	high	25	16.45%	8	18.18%	19	27.54%	5	22.73%		
GSES	low	14	9.21%	9	20.45%	6	8.70%	0	0.00%	0.157	0.127
	average	47	30.92%	14	31.82%	23	33.33%	10	45.45%		
	high	91	59.87%	21	47.73%	40	57.97%	12	54.55%		

Variable		Place of work				CH	Cramer’s V
		Hospital for adult patients		Pediatric hospital			
SWLS	low	28	40.58%	100	45.87%	0.737	0.046
	average	32	46.38%	91	41.74%		
	high	9	13.04%	27	12.39%		
CISS SSZ	low	26	37.68%	81	37.16%	0.646	0.055
	average	33	47.83%	95	43.58%		
	high	10	14.49%	42	19.27%		
CISS SSE	low	15	21.74%	60	27.52%	0.127	0.120
	average	40	57.97%	96	44.04%		
	high	14	20.29%	62	28.44%		
CISS SSU	low	12	17.39%	60	27.52%	0.135	0.118
	average	38	55.07%	93	42.66%		
	high	19	27.54%	65	29.82%		
CISS SSU ACZ	low	10	14.49%	48	22.02%	0.398	0.080
	average	31	44.93%	90	41.28%		
	high	28	40.58%	80	36.70%		
CISS SSU PKT	low	16	23.19%	71	32.57%	0.121	0.121
	average	34	49.28%	109	50.00%		
	high	19	27.54%	38	17.43%		
GSES	low	6	8.70%	23	10.55%	0.905	0.026
	average	23	33.33%	71	32.57%		
	high	40	57.97%	124	56.88%		

SWLS, Life Satisfaction; CESS, Emotional Control Level; CISS, Stress Coping Style; CISS SSZ, Task-oriented Coping; CISSSSE, Emotion-oriented Coping. CISS SSU, Avoidance-oriented Coping; CISSSUACZ, Avoidance-oriented Coping, Engaging in Substitute Activities; CISSSSUPKT, Avoidance-oriented Coping, Seeking Social Contacts. GSES, Self-Efficacy; SLIFS, Death Anxiety and Fascination Scale.

The level of emotional control, as well as death anxiety and fascination, did not vary significantly according to the respondents’ place of work (Table 4).

**Table 4:** Relationships between scale scores and respondents’ place of work.

Variable	Hospital	n	Min.	Max.	Median	Q1	Q3	Mean	SD	
CECS anger scale	Biała Podlaska	152.00	7	28	18	14	21	17.6	4.51	ANOVA *p* = 0.652
	Białystok	44.00	7	26	16	13	21	16.7	4.75	
	Lublin	69.00	7	28	17	14	20	17.2	4.28	
	Warsaw	22.00	8	25	16.5	15	19	16.9	4.65	
CECS depression scale	Biała Podlaska	152.00	7	28	18	15	20	17.4	4.03	ANOVA *p* = 0.889
	Białystok	44.00	8	28	18	15.8	20	17.6	4.36	
	Lublin	69.00	7	27	18	15	20	17.8	4.1	
	Warsaw	22.00	10	22	17	16	19	17.4	2.9	
CECS anxiety scale	Biała Podlaska	152.00	7	28	18	16	19	17.9	3.76	Kruskal- Wallis *p* = 0.8005
	Białystok	44.00	7	28	18	16.8	21.2	18.6	4.57	
	Lublin	69.00	10	28	18	16	19	18.3	3.47	
	Warsaw	22.00	11	25	17.5	16	19.8	17.9	3.45	
CECS overall index of emotional control	Biała Podlaska	152.00	21	77	53	47	58.2	52.8	9.56	ANOVA *p* = 0.968
	Białystok	44.00	30	81	53	46.8	60.5	52.9	11.2	
	Lublin	69.00	30	79	53	48	59	53.3	9.47	
	Warsaw	22.00	37	68	51.5	46.2	56.8	52.2	8.54	
SLIFS	Biała Podlaska	152.00	23	92	47	43	51.2	47.9	9.33	Kruskal- Wallis *p* = 0.1653
	Białystok	44.00	29	69	44	40.8	49.2	45.1	6.94	
	Lublin	69.00	24	58	47	42	49	45.2	6.48	
	Warsaw	22.00	31	92	46	42.5	50	48.8	13	
SLIFS death fascination subscale	Biała Podlaska	152.00	11	44	21	18	23	21.2	5.09	Kruskal- Wallis *p* = 0.2016
	Białystok	44.00	12	33	19.5	17	21	19.6	3.54	
	Lublin	69.00	11	27	20	17	23	19.8	3.68	
	Warsaw	22.00	14	44	20	18.2	23	21.7	6.88	
SLIFS death anxiety subscale	Biała Podlaska	152.00	9	36	18	17	21	18.7	3.88	Kruskal- Wallis *p* = 0.3621
	Białystok	44.00	9	27	18	15	20	17.8	3.38	
	Lublin	69.00	10	24	18	16	19	17.9	2.82	
	Warsaw	22.00	10	36	18.5	17	20.8	19.2	5.6	

CESS, Emotional Control; SLIFS, Death Anxiety and Fascination Scale.

With age, respondents obtained significantly higher levels of life satisfaction and task-oriented and emotion-oriented coping. The same correlation was observed for the respondents’ length of service in the context of life satisfaction and task-oriented coping. The level of avoidance-oriented coping, including mainly engaging in substitute activities, correlated significantly negatively with the age and length of service of the respondents (Table 5).

**Table 5:** Correlations between questionnaire results and respondents’ age and length of service (Kendall’s Tau correlation coefficient).

Variable	Age		Length of service	
	*p*	Tau	*p*	Tau
SWLS	0.011	0.132	0.024	0.116
CISS SSZ	0.002	0.157	0.025	0.115
CISS SSE	0.015	0.125	0.052	0.099
CISS SSU	0.000	−0.204	0.001	−0.166
CISS SSU ACZ	0.003	−0.154	0.003	−0.152
CISS SSU PKT	0.288	−0.055	0.734	−0.017
GSES	0.876	0.008	0.503	0.035
CECS anger scale	0.735	0.016	0.877	−0.007
CECS depression scale	0.181	0.061	0.277	0.049
CECS anxiety scale	0.193	−0.060	0.050	0.090
Overall index of emotional control	0.944	0.003	0.441	−0.034
SLIFS total	0.099	0.075	0.437	0.035
SLIFS death fascination subscale	0.297	0.048	0.634	0.022
SLIFS death anxiety	0.114	0.073	0.491	0.031

SWLS, Life Satisfaction; CESS, Emotional Control Level; CISS, Stress Coping Style; CISS SSZ, Task-oriented Coping; CISSSSE, Emotion-oriented Coping. CISS SSU, Avoidance-oriented Coping; CISSSUACZ, Avoidance-oriented Coping, Engaging in Substitute Activities; CISSSSUPKT, Avoidance-oriented Coping, Seeking Social Contacts. GSES, Self-Efficacy; SLIFS, Death Anxiety and Fascination Scale.

No significant relationship was observed between a life satisfaction level and showing a dying patient compassion based on the patient’s position in society. Also, after taking the age, job specifics, length of service and place of work into consideration, no such relationship was observed (Table 6).

**Table 6:** The relationship between showing a dying patient compassion based on the patient’s position in society and the SWLS questionnaire score taking age, job specifics, length of service and place of work into consideration.

	SWLS sten score					
	Taking the age and job specifics (children/adults) into consideration			Taking the length of service and place of work into consideration		
	Coefficient beta	Standard error	Pr	Beta coefficient	Standard error	Pr
I treat all people equally	−0.419	0.239	0.081	−0.420	0.241	0.083
It varies	−0.502	0.366	0.171	−0.587	0.373	0.117

No significant relationship was observed between the following: task-oriented coping, emotion-oriented coping, avoidance-oriented coping and engaging in substitute activities, avoidance-oriented coping and seeking social contact, and avoidance-oriented coping style expressed as the sten scores in stress coping and showing a dying patient compassion depending on the patient’s position in society. Also, after taking the age, job specifics, length of service and place of work into consideration, no such relationship was observed (Table 7).

**Table 7:** The relationship between showing a dying patient compassion based on the patient’s position in society and the CIS questionnaire score taking age, job specifics, length of service and place of work into consideration.

	Taking the age and job specifics (children/adults) into consideration			Taking the length of service and place of work into consideration		
	Beta coefficient	Standard error	Pr	Beta coefficient	Standard error	Pr
CISS SSZ sten score						
I treat all people equally	0.491	0.261	0.061	0.476	0.262	0.071
it varies	0.102	0.399	0.799	0.079	0.405	0.846
CISS SSE sten score						
I treat all people equally	−0.239	0.266	0.371	−0.234	0.263	0.375
it varies	0.515	0.408	0.208	0.623	0.408	0.128
CISS SSU ACZ sten score						
I treat all people equally	−0.101	0.273	0.712	−0.075	0.274	0.785
it varies	0.410	0.418	0.328	0.574	0.424	0.177
CISS SSU PKT sten score						
I treat all people equally	0.024	0.261	0.927	0.051	0.265	0.849
it varies	0.136	0.400	0.734	0.121	0.409	0.768
CICC SSU sten score						
I treat all people equally	−0.256	0.257	0.321	−0.237	0.261	0.366
it varies	0.232	0.394	0.556	0.369	0.404	0.362

No significant relationship was observed between the level of subjective emotional control, depression, anxiety and the Overall Index of Emotional Control in difficult situations and showing a dying patient compassion based on the patient’s position in society. Also, after taking the age, job specifics, length of service and place of work into consideration, no such relationship was observed (Table 8).

**Table 8:** The relationship between showing a dying patient compassion based on the patient’s position in society and the CECS questionnaire score, taking age, job specifics, length of service and place of work into consideration.

	Taking the age and job specifics (children/adults) into consideration			Taking the length of service and place of work into consideration		
	Beta coefficient	Standard error	Pr	Beta coefficient	Standard error	Pr
CECS anger scale						
I treat all people equally	0.197	0.639	0.758	0.226	0.644	0.726
it varies	0.064	0.980	0.948	0.128	0.995	0.898
CESS depression scale						
I treat all people equally	−0.478	0.570	0.402	−0.352	0.573	0.540
it varies	0.791	0.874	0.366	0.775	0.887	0.383
CESS anxiety scale						
I treat all people equally	−0.708	0.534	0.186	−0.715	0.536	0.183
it varies	0.081	0.819	0.921	0.019	0.828	0.982
Overall index of emotional control						
I treat all people equally	−0.989	1.379	0.474	−0.841	1.386	0.545
it varies	0.936	2.114	0.658	0.921	2.144	0.668

No significant relationship was observed between the level of life satisfaction and perceiving the death of patients from different age groups as harrowing. Also, after taking the age, job specifics, length of service and place of work into consideration, no such relationship was observed (Table 9).

**Table 9:** The relationship between perceiving the death of patients from different age groups as harrowing to various degrees and the SWLS questionnaire score, taking the age, job specifics, length of service and place of work into consideration.

SWLS sten score						
	Taking the age and job specifics (children/adults) into consideration			Taking the length of service and place of work into consideration		
	Beta coefficient	Standard error	CH	Beta coefficient	Standard error	CH
Children/adolescents	0.321	0.313	0.306	0.262	0.313	0.404
Adults	0.444	0.594	0.455	0.335	0.595	0.574
Any	0.125	0.225	0.579	0.124	0.230	0.590

No significant relationship was observed between task-oriented, emotion-oriented and avoidance-oriented coping and perceiving the death of patients from each age group as harrowing. However, after taking the age and job specifics into consideration, it was observed that some study subjects choosing the “any kind of death” response had a significantly lower score than the other respondents. Taking the length of service and place of work into consideration, the respondents who chose “death of adolescents” and “any death” had significantly lower questionnaire scores. The respondents for whom the death of adults was most shocking were significantly more likely to follow the avoidance-oriented coping in engaging in substitute activities than those for whom the death of adolescents or any death was more harrowing. The relationship is no longer relevant when taking the age and job specifics into consideration. On the other hand, when taking the length of service and place of work into consideration, only one correlation was observed: significantly more respondents who chose the answer “death of adults” followed avoidance-oriented coping—engaging in substitute activities (Table 10).

**Table 10:** The relationship between perceiving the death of patients from different age groups as harrowing to various degrees and the CISS questionnaire score, taking the age, job specifics, length of service and place of work into consideration.

	Taking the age and job specifics (children/adults) into consideration			Taking the length of service and place of work into consideration		
CISS SSZ sten score						
	Beta coefficient	Standard error	CH	Beta coefficient	Standard error	CH
Children/adolescents	−0.583	0.341	0.088	−0.682	0.340	0.046
Adults	−0.576	0.647	0.374	−0.762	0.646	0.239
Any	−0.498	0.245	0.043	−0.580	0.249	0.021
CISS SSE sten score						
Children/adolescents	−0.075	0.349	0.831	−0.037	0.342	0.913
Adults	0.741	0.662	0.264	0.628	0.651	0.335
Any	0.191	0.251	0.447	0.231	0.251	0.358
CISS SSU ACZ sten score						
Children/adolescents	−0.341	0.357	0.341	−0.186	0.356	0.600
Adults	1.204	0.678	0.077	1.401	0.676	0.039
Any	−0.226	0.257	0.380	−0.096	0.261	0.713
CISS SSU PKT sten score						
Children/adolescents	−0.550	0.340	0.107	−0.668	0.340	0.051
Adults	0.051	0.645	0.937	0.074	0.647	0.909
Any	−0.165	0.244	0.500	−0.193	0.250	0.440
CISS SSU sten score						
Children/adolescents	−0.430	0.339	0.206	−0.373	0.340	0.273
Adults	0.803	0.643	0.213	0.972	0.646	0.134
Any	−0.265	0.243	0.276	−0.185	0.249	0.460

No significant relationship was observed between the presented level of emotional control in the anger, depression and anxiety scales and the overall emotional control index, and perceiving the death of patients from different age groups as harrowing to various degrees. Also, after taking the age, job specifics, length of service and place of work into consideration, no such relationship was observed (Table 11).

**Table 11:** The relationship between perceiving the death of patients from different age groups as harrowing to various degrees and the CECS questionnaire score, taking the age, job specifics, length of service and place of work into consideration.

	Taking the age and job specifics (children/adults) into consideration			Taking the length of service and place of work into consideration		
	Beta coefficient	Standard error	CH	Beta coefficient	Standard error	CH
CECS anger scale						
Children/adolescents	−0.630	0.833	0.450	−0.521	0.831	0.531
Adults	−0.887	1.581	0.575	−1.265	1.578	0.424
Any	−0.209	0.599	0.727	−0.129	0.610	0.832
CECS depression scale						
Children/adolescents	0.385	0.748	0.607	0.513	0.743	0.491
Adults	0.985	1.419	0.488	0.928	1.413	0.512
Any	0.297	0.537	0.581	0.308	0.546	0.573
CECS anxiety scale						
Children/adolescents	−0.422	0.692	0.543	0.030	0.688	0.966
Adults	0.111	1.314	0.933	0.492	1.308	0.707
Any	0.973	0.497	0.051	1.041	0.505	0.040
Overall index of emotional control						
Children/adolescents	−0.668	1.799	0.711	0.022	1.789	0.990
Adults	0.209	3.415	0.951	0.155	3.400	0.964
Any	1.060	1.293	0.413	1.220	1.313	0.354

No significant relationship was observed between the respondents’ understanding of death and their life satisfaction level, task-oriented coping, emotion-oriented coping, avoidance-oriented coping with engaging in substitute activities, by seeking social contact, and the level of depression control, anxiety control and overall emotional control index. Also, after taking the age, job specifics, length of service and place of work into consideration, no such relationship was observed It was observed that the respondents who regarded death as a defeat in the struggle for life had a significantly higher level of anger control than those who regarded death as the end of suffering and pain. After taking the age and job specifics as well as the length of service and location into consideration, it was shown that the work of those who considered death as a defeat in the struggle for life achieved significantly higher levels of anger control (Table 12).

**Table 12:** The relationship between respondents’ understanding of death and SWLS, CISS, and CESS questionnaire scores.

	Taking the age and job specifics (children/adults) into consideration			Taking the length of service and place of work into consideration		
	Beta coefficient	Standard error	CH	Beta coefficient	Standard error	CH student’s *t*-test
SWLS sten score						
End of suffering and pain	0.113	0.224	0.614	0.146	0.229	0.524
Defeat in the struggle for life	0.288	0.311	0.355	0.345	0.313	0.271
CISS SSZ sten score						
End of suffering and pain	0.045	0.246	0.856	0.047	0.252	0.852
Defeat in the struggle for life	−0.299	0.343	0.384	−0.264	0.344	0.444
CISS SSE sten score						
End of suffering and pain	0.486	0.250	0.053	0.528	0.251	0.036
Defeat in the struggle for life	0.007	0.348	0.985	0.056	0.342	0.871
CISS SSU ACZ sten score						
End of suffering and pain	0.144	0.258	0.577	0.161	0.263	0.541
Defeat in the struggle for life	−0.030	0.359	0.934	−0.110	0.359	0.759
CISS SSU points						
End of suffering and pain	−0.208	0.245	0.397	−0.209	0.251	0.407
Defeat in the struggle for life	−0.291	0.341	0.394	−0.442	0.343	0.199
CISS SSU sten score						
End of suffering and pain	−0.085	0.245	0.729	−0.080	0.252	0.752
Defeat in the struggle for life	−0.105	0.341	0.759	−0.234	0.344	0.496
CECS anger scale						
End of suffering and pain	−0.763	0.588	0.195	−0.848	0.597	0.157
Defeat in the struggle for life	1.704	0.818	0.038	1.904	0.815	0.020
CECS depression scale						
End of suffering and pain	0.099	0.537	0.853	−0.001	0.546	0.998
Defeat in the struggle for life	0.237	0.747	0.751	0.160	0.746	0.831
CECS anxiety scale						
End of suffering and pain	0.083	0.502	0.869	−0.111	0.508	0.828
Defeat in the struggle for life	0.220	0.698	0.753	0.393	0.693	0.572
Overall index of emotional control						
End of suffering and pain	−0.581	1.284	0.651	−0.959	1.300	0.461
Defeat in the struggle for life	2.161	1.787	0.227	2.456	1.776	0.168

The interrelationships between life satisfaction, stress coping styles, levels of generalized self-efficacy, emotional control styles, and Death Anxiety and Fascination Scale were also analyzed. The results are shown in Table 13.

**Table 13:** Correlations of questionnaire results.

	SWLS sten	CISS SSZ sten score	CISS SSE sten score	CISS SSU sten score	CISS SSU Acz sten score	CISS SSU PKT sten score	GSES sten score	CECS overall index	CECS anger scale	CECS depression scale	CECS anxiety scale	SLIFS total	SLIFS death fascination subscale	SLIFS death anxiety
														Death anxiety subscale
SWLS sten	0.000	0.806	0.000	0.366	0.736	0.003	0.001	0.601	0.381	0.038	0.850	0.776	0.840	0.612
CISS SSZ sten score	0.806	0.000	0.184	0.041	0.418	0.000	0.000	0.239	0.941	0.219	0.105	0.495	0.073	0.964
CISS SSE sten score	0.000	0.184	0.000	0.000	0.000	0.020	0.000	0.103	0.669	0.001	0.301	0.000	0.000	0.000
CISS SSU sten score	0.366	0.041	0.000	0.000	0.000	0.000	0.542	0.853	0.860	0.902	0.892	0.002	0.006	0.005
CISS SSU Acz sten score	0.736	0.418	0.000	0.000	0.000	0.000	0.595	0.316	0.700	0.125	0.629	0.000	0.000	0.000
CISS SSU point/sten score	0.003	0.000	0.020	0.000	0.000	0.000	0.058	0.048	0.203	0.020	0.275	0.814	0.965	0.927
GSES sten score	0.001	0.000	0.000	0.542	0.595	0.058	0.000	0.349	0.378	0.017	0.359	0.018	0.010	0.013
CECS overall index	0.601	0.239	0.103	0.853	0.316	0.048	0.349	0.000	0.000	0.000	0.000	0.682	0.480	0.789
CECS Anger Scale	0.381	0.941	0.669	0.860	0.700	0.203	0.378	0.000	0.000	0.000	0.000	0.678	0.663	0.183
CECS depression scale	0.038	0.219	0.001	0.902	0.125	0.020	0.017	0.000	0.000	0.000	0.000	0.214	0.325	0.421
CECS anxiety scale	0.850	0.105	0.301	0.892	0.629	0.275	0.359	0.000	0.000	0.000	0.000	0.821	0.806	0.967
SLIFS total	0.776	0.495	0.000	0.002	0.000	0.814	0.018	0.682	0.678	0.214	0.821	0.000	0.000	0.000
SLIFS death fascination subscale	0.840	0.073	0.000	0.006	0.000	0.965	0.010	0.480	0.663	0.325	0.806	0.000	0.000	0.000
SLIFS death anxiety death anxiety subscale	0.612	0.964	0.000	0.005	0.000	0.927	0.013	0.789	0.183	0.421	0.967	0.000	0.000	0.000

In the case of the majority (84.67%) of the respondents, the employer did not provide mental support for the staff in difficult situations, but 79.44% felt that such assistance in their work was important, and half of the respondents (52.26%) would welcome it.

The majority of the respondents did not feel the need to broaden their knowledge of death and dying (64.11%) and had never been to a training course devoted to such topics (78.75%). However, 70.73% of the respondents would attend such a course, if it were available. The most common sources of knowledge of how to deal with a dying patient were the press and the Internet (39.72%), a therapeutic team (15.68%), training (14.98%), and church (14.29%). 13.94% of the respondents were convinced that such knowledge was unnecessary for them, and 1.39% had no opinion on the matter. According to the respondents, the most stressful factors at work included the relationship with a divisional nurse (mean score of 3.37 ± 1.5) and the relationship with a patient (mean score of 3.1 ± 1.54), relationship with physicians (mean score of 3.02 ± 1.17), patient death (mean score of 3.01 ± 1.54), and job specifics (mean score of 2.47 ± 1.5).

Thanks to the verification of the formulated hypotheses, we could conclude that hypotheses 1, 3, 5, and 7 were partially confirmed, hypothesis 2 was not confirmed, and hypotheses 4, 6 and 8 were confirmed.

## Discussion

In the present study, the surveyed nurses most often presented low or average life satisfaction, high self-efficacy, average levels of stress and coping in all three styles (with a preference for avoidance-oriented), and a high level of death fascination and an average level of death anxiety. The majority of the nurses in difficult situations and when a stressful situation occurred did not receive support from their employer or from physicians but could count on the assistance of fellow nurses and a divisional nurse. The majority of the respondents felt that psychological support in their work was important and would gladly benefit from it. Although most nurses did not feel the need to broaden their knowledge of death/dying, they would take part in such a course if it were possible.

Our findings are in accordance with previous reports (2, 22–25).

In a study by Guzowski et al. (22), according to respondents, dying is an inevitability and suffering, and a good death is a death without pain, while a bad death is a death in solitude (60% of people). The word “death” was associated by the respondents primarily (50%) with old age, and hearing it, they were most often filled with (71%) contemplation and reflection. This was also confirmed by the current study, as 54% of nurses considered death to be a natural process like birth, and they tended to associate the word “death” with passing away and inevitability (56.3%).

A 2018 systematic review (23) included 10 of 16 qualitative studies (23). These studies evaluated intrinsic and extrinsic resources. The intrinsic resources consisted of setting boundaries, reflection, crying, death beliefs, life and work experience, and daily routines and activities. The extrinsic resources were comprised of talking and being heard, spiritual practices, education and programs, and debriefing. They concluded that nurses need more support resources, which better assist them in coping with patient death.

A more recent systematic review from 2020 (24) included 17 articles. Thirteen categories emerged, which were grouped into three themes: meanings and feelings during the dying process, coping strategies in the face of death, and the importance of training, experience, and providing a dignified death. It was found that death had a large negative emotional impact on them. The participants complained about the lack of previous training in the care of dying patients. The authors concluded that the lack of training in the basic care of terminally ill patients and negative ideas about death both cause health professionals to experience situations of great stress and frustration, resulting, on many occasions, in resorting to avoidance of these situations, thus preventing dying with dignity.

A recent multicenter (Australia, China), cross-sectional study from 2021 (25) examined 340 new graduate nurses’ perceptions of competency in coping with dying and death and the relationship with death self-efficacy and death anxiety of new graduate nurses. 88.9% feared a painful death, 81.5% were particularly afraid of getting cancer, and 80.2% were afraid of death. There was a positive correlation between coping with death and death self-efficacy, a negative relationship between coping with death and death anxiety, and a negative relationship between death self-efficacy and death anxiety. The authors concluded that new graduate nurses are at a disadvantage in terms of death self-efficacy, are less well-prepared to cope with death, and are more anxious about death.

Merklinger-Soma et al. (23) distinguished three types of attitudes accompanying them during contact with death: a neutral one, a distanced one, and an emotional one. According to the authors, the elements of a perfect attitude toward death are represented in the neutral type. Nyklewicz and Krajewska-Kułak (24) demonstrated that 73% of nurses rated their depression toward the death of a loved one and their fear of the death of a loved one (69%) as very high. The majority of the respondents (92.8%) surveyed by Guzowski et al. (22) were convinced that the perception of death could also be influenced by religion and experiencing the death of a loved one. The respondents were more likely to think about the death of their loved ones (16%) than their own death (13.3%). In contrast, this study showed that a significantly higher percentage of the nurses (67.3%) thought about their own death, a higher percentage (78.4%) thought about the death of their loved ones, 56.8% about the death of strangers, and 50.5% about the death of their friends. In their study involving 114 nurses, Krajewska-Kułak et al. (25) found that during contact with dying patients, they often felt sadness (38.6%) and helplessness (32.5%). The respondents did not usually avoid contact with dying patients (51.8%), accepted death as a natural phenomenon (40.4%), did not feel anxiety (36%), but also did not accept death, stating that it was absolutely necessary to struggle for a patient’s life until the very end (34%). Similarly, Mickiewicz, et al. (26) also showed that 37.7% of nurses felt sadness, and 33.3% felt helplessness during the death of a patient. Also, Niedojad et al. (2) found that the most common reactions accompanying the nursing staff in the face of a patient’s death were feeling sadness (82%), accepting death peacefully (75%), and being horrified at the prevailing injustice (52%). In the study by Uchmanowicz et al. (27) 80% of the nurses involved felt depressed and sad about the death of their patients. Also, Niedojad et al. (2) found that the most common emotions accompanying nurses in the face of a patient’s death included compassion and sadness (91%), grief, and depression (70%). Similarly, the nurses involved in the study by Ponińska and Chojnacka-Kowalewska (28) most often felt sadness (65%) after a patient’s death. Analysis of the results from the study conducted by Głowacka et al. (1) also revealed that 82.7% of the respondents in the situation of a patient’s death most often felt sadness, 74% felt compassion, 56% felt powerlessness, and 40% felt helplessness (40%). Anxiety appeared in 20% of the respondents, and 10% of the nurses involved in the study felt relief in the situation of a patient’s death (1), which correlated with Zawiślak’s study (29). Sadness was among the most common emotions (39.3%).

It is worth pointing out that in the case of healthcare professionals, contact with death is much more frequent than for other people, and during the first clinical activities, they start witnessing the death of their patients, so they may feel anxiety and discomfort about it. Performing this very difficult task of accompanying a dying patient, nurses can, as mentioned before, adopt many different attitudes toward their patients (27, 28).

Literature data (25, 26), suggest that the age of nurses significantly influences the degree of anxiety associated with the death of a patient. Less anxiety and greater acceptance of standard procedures for caring for dying patients were associated with older nurses. Working with dying patients was reported as stressful by 79% of the nurses with many years of service, compared to the group with short work experience. The current study demonstrated that with age and length of service, nurses reached significantly higher levels of life satisfaction and task-oriented coping. In contrast, avoidance-oriented coping, including mainly engaging in substitute activities, correlated negatively with the age and length of service of the respondents. There was also no relationship between the age and length of service and the position in society of a dying patient and showing them compassion, or perception of death as harrowing.

Death anxiety seems to be a natural reaction to danger, but unfortunately, when it is strong and absorbing, it may hinder undertaking professional and everyday tasks. Rababa et al. (30) demonstrated that high levels of death anxiety and low levels of self-esteem, interpersonal reactivity, and symbolic immortality were associated with increased levels of ageism among nurses. According to Kostka et al. (31), the nurses who have frequent contact with dying and death in their daily professional practice show less anxiety compared to the nurses who have such contact occasionally.

The literature (32, 33) emphasizes that death anxiety can be considered a universal phenomenon that appears relatively early in human psychological development but is heterogeneous. It can affect oneself or others or have an abstract meaning. This study assessed a so-called level of death fascination and anxiety using the standardized Death Anxiety and Fascination Scale. The Death Anxiety Subscale is used to measure a conscious level of death anxiety, especially in relation to oneself, whereas the higher the death anxiety, the more cruel, dark, and dirty it is perceived to be. The Death Fascination Subscale measures the level of death fascination, understood as cognitive interest in the topics of death and dying, whereas the higher the fascination, the more death is perceived as pure and merciful. The nurses involved in this study presented a high level of death fascination and average levels of anxiety.

Kucharewicz and Stochmiałek (34) found a specific relationship between death anxiety and level of religiosity: the more respondents experienced death anxiety, the more important religious practices were to them. In a sense, it was reflected in this study, as 33.8% of the nurses regarded prayer as the greatest help in dealing with the difficulties of experiencing a patient’s death. On the other hand, Nyklewicz and Krajewska-Kułak (24) showed that while thinking about death, as many as 74% of the respondents experienced above-average anxiety. In another study, the same authors found that as many as 71% of the nursing students reacted to death with despondency, 45% had depressive disorders, and 49% had general health disorders. Above-average high death anxiety was found in 72% of the students.

Preventing and coping with the negative effects of stress is undoubtedly a prerequisite for job satisfaction. In the study by Ponińska and Chojnacka-Kowalewska (29), the nurses rated their stress related to the death of a patient as medium (57%) or high (32%). Nyklewicz and Krajewska-Kułak (24) found that 74% of nurses experienced above-average death anxiety. Unfortunately, as many as 78% of the nurses ineffectively reacted to stress through anger suppression (69%), depression (72%) and anxiety (76%). Task-oriented coping was observed in 22% of the nurses, and emotion-oriented and avoidance-oriented coping was observed in 9% of the respondents. As many as 60% of the respondents did not use any of the discussed coping styles in a dominant way (24). Different results were obtained in this study, as the respondents presented generally average stress coping levels in all three styles (including the highest for avoidance-oriented coping). A level of avoidance-oriented coping, including mainly engaging in substitute activities correlated significantly negatively with the respondents’ age and length of service, while task-oriented coping increased with the length of service.

Following Strelau et al. (19), nurses’ task-oriented coping suggests that they may try to solve problems by trying to change the situation or transform it in a cognitive way. Emotion-oriented coping means that in stressful situations, the individuals focus on themselves, on their own emotional experiences, such as anger, guilt, and tension, accompanied by wishful thinking and fantasizing, unfortunately not leading to active problem-solving. Such individuals are characterized by low social skills, low emotional intelligence, and higher levels of neuroticism (19). Avoidance-oriented coping, dominant in this study, involves shying away from thinking about, encountering, and experiencing certain stressful situations. Such individuals are more likely to engage in substitute activities, such as sleeping, watching TV, overeating, thinking about pleasant things, and seeking the company of other people.

According to 80% of the nursing staff surveyed by Uchmanowicz et al. (27), they are adequately prepared to work with a dying patient. Although the majority of the individuals surveyed in this study (64.1%) did not feel the need to broaden their knowledge of death/dying, as many as 70.7% of nurses would benefit from a course devoted to this topic. As a profession that requires emotional involvement in other people’s affairs, nursing is, unfortunately, strictly connected with the risk of the appearance of the lack of distance from patients’ problems. The consequence of the above may be experiencing profound patient concerns, including even treating them as one’s own, disrupting the image and order of the employees’ world, causing instability, gradual loss of energy, and, finally, developing professional burnout. Unfortunately, nurses often tend to accumulate negative emotions. Furthermore, 90% of the surveyed nurses declared suffering from work-related stress, which was correlated mainly with sleep problems, inability to focus, and lack of strength, energy, and motivation to continue working.

Modzelewska and Kulik (11) attempted to assess nurses’ need for professional support when experiencing stress and symptoms of professional burnout syndrome. All the respondents showed interest in such a form of support, and 86.7% of them said they were willing to participate in group meetings to support them in coping with stress and emotional exhaustion. In this current study, most nurses would benefit from such a course if possible, although they did not feel the need to deepen their knowledge of death/dying.

### Study limitations

Our study has some limitations. The study is not a randomized study, which could affect bias selection. When certain groups are more likely to be included in the study due to non-random sampling. Participants have different abilities to recall past events or experiences. Nurses worked in different departments (surgery, anaesthesiology, oncology, gynecology, geriatrics, cardiology, internal medicine, and neonatology). Nurses’ experience in these departments may affect their ability to cope with death. Also, the age of nurses could impact on coping styles. Most respondents were aged between 41 and 60.

### Implications

It seems necessary to enable nurses to work through the emotions resulting from contact with dying patients with a psychotherapist or psychologist who will direct them to the correct perception of death. Hospitals and medical centers should contribute to social support and psychological assistance along with training provided on stress-coping strategies for nurses.

We suggest that future research should be performed with a larger study group and additional factors influencing the perception of death and dying, for example, religion or belief and the number of deaths.

## Conclusion

1. The surveyed nurses most often presented low or average life satisfaction, high self-efficacy, average levels of stress and coping in all three styles (with a preference for avoidance-oriented), and a high level of death fascination and an average level of death anxiety.
2. Showing compassion for a dying patient regardless of their position in society, and perceiving the death of patients from different age groups as harrowing did not depend on the nurses’ age, job specifics, years of service, level of life satisfaction, stress coping style and level of emotional control.
3. The respondents for whom the death of adults seemed the most harrowing were significantly more likely than others to present an avoidance-oriented coping style in engaging in substitute activities.
4. The majority of the nurses in difficult situations and when a stressful situation occurred did not receive support from their employer or from physicians but could count on the assistance of fellow nurses and a divisional nurse.
5. The majority of the respondents felt that psychological support in their work was important and would gladly benefit from it.
6. Although most nurses did not feel the need to broaden their knowledge of death/dying, they would take part in such a course if it were possible.

## Data availability statement

The raw data supporting the conclusions of this article will be made available by the authors, without undue reservation.

## Ethics statement

The studies involving humans were approved by the Bioethics Committee R-I-002/631/2019, the Medical University of Bialystok. The studies were conducted in accordance with the local legislation and institutional requirements. The participants provided their written informed consent to participate in this study.

## Author contributions

MK: Conceptualization, Data curation, Investigation, Writing – original draft. EK-K: Conceptualization, Formal analysis, Methodology, Supervision, Writing – review & editing. BK: Supervision, Writing – review & editing. AK-B: Supervision, Writing – review & editing. TK: Writing – review & editing. AG-K: Supervision, Writing – review & editing. KD-O: Supervision, Writing – review & editing.
